# Integrative Assessments on Molecular Taxonomy of *Acidiferrobacter thiooxydans* ZJ and Its Environmental Adaptation Based on Mobile Genetic Elements

**DOI:** 10.3389/fmicb.2022.826829

**Published:** 2022-02-16

**Authors:** Liyuan Ma, Weiyi Yang, Shanshan Huang, Rui Liu, Huiying Li, Xinping Huang, Junming Xiong, Xueduan Liu

**Affiliations:** ^1^Hubei Key Laboratory of Yangtze Catchment Environmental Aquatic Science, School of Environmental Studies, China University of Geosciences, Wuhan, China; ^2^Key Laboratory of Biometallurgy of Ministry of Education, Central South University, Changsha, China

**Keywords:** *Acidiferrobacter*, complete genome, comparative genomics, genome plasticity, environmental adaptation

## Abstract

Acidiferrobacter spp. are facultatively anaerobic acidophiles that belong to a distinctive *Acidiferrobacteraceae* family, which are similar to *Ectothiorhodospiraceae* phylogenetically, and are closely related to *Acidithiobacillia* class/subdivision physiologically. The limited genome information has kept them from being studied on molecular taxonomy and environmental adaptation in depth. Herein, *Af. thiooxydans* ZJ was isolated from acid mine drainage (AMD), and the complete genome sequence was reported to scan its genetic constitution for taxonomic and adaptative feature exploration. The genome has a single chromosome of 3,302,271 base pairs (bp), with a GC content of 63.61%. The phylogenetic tree based on OrthoANI highlighted the unique position of *Af. thiooxydans* ZJ, which harbored more unique genes among the strains from *Ectothiorhodospiraceae* and *Acidithiobacillaceae* by pan-genome analysis. The diverse mobile genetic elements (MGEs), such as insertion sequence (IS), clustered regularly interspaced short palindromic repeat (CRISPR), prophage, and genomic island (GI), have been identified and characterized in *Af. thiooxydans* ZJ. The results showed that *Af. thiooxydans* ZJ may effectively resist the infection of foreign viruses and gain functional gene fragments or clusters to shape its own genome advantageously. This study will offer more evidence of the genomic plasticity and improve our understanding of evolutionary adaptation mechanisms to extreme AMD environment, which could expand the potential utilization of *Af. thiooxydans* ZJ as an iron and sulfur oxidizer in industrial bioleaching.

## Introduction

The type strain *Acidiferrobacter thiooxydans* m-1 was initially reported as “*Thiobacillus ferrooxidans*” owing to its iron-oxidizing capability, which was isolated from coal spoil refuse in Missouri, United States, over 35 years ago (Harrison, 1982). The representative genus *Acidithiobacillus* (formerly *Thiobacillus*) was the most widespread one with higher Fe/S oxidation ability, such as *A. ferrooxidans*, *A. ferrivorans*, *A. caldus*, and *A. thiooxidans* (Ma et al., 2021). However, the strain *Af. thiooxydans* m-1 harbored a higher chromosomal GC content and the analysis of partial 16S rRNA sequences revealed that it was quite distantly related to other acidophilic bacteria. This strain remained poorly studied on the physiology and phylogeny until 2011. Researchers renamed the strain as *Af. thiooxydans* and confirmed that it was closely related with the members of the family *Ectothiorhodospiraceae*, order *Chromatiales*, also including typically alkaliphilic and halophilic bacteria that could not oxidize iron and sulfur (Hallberg et al., 2011). Another member within the genus, *Acidiferrobacter* sp. strain SP-III/3 (DSM 27195), emerged as a new clade from the 16S rRNA-based phylogeny, which was isolated from AMD in Cartagena (Murcia, Spain) (Mitchell et al., 2004). The energy metabolism strategies from this genus, such as iron and sulfur oxidation features, were deeply analyzed *via* comparative genomics (Issotta et al., 2018). However, the calculated ANI relatedness between SPIII/3 and m-1 was lower than 95%, implying that they could not be assigned to the same species obviously.

In 2015, a novel chemolithoautotrophic sulfur oxidizer, called *Sulfurifustis variabilis* skN76, was isolated and characterized from the sediment of Lake Mizugaki (Kojima et al., 2015). It was closely related to *Af. thiooxydans*, but was distinct from other members within the family *Ectothiorhodospiraceae*. They were assigned into a novel family *Acidiferrobacteraceae* affiliated to the order *Acidiferrobacterales*. A year later, a similar novel sulfur oxidizer *Sulfuricaulis limicola* HA5, which was isolated from the sediment of Lake Harutori, was also established and assigned to the family *Acidiferrobacteraceae* (Kojima et al., 2016). Redundancies of the sulfite reduction and oxidation genes were observed in both of the two complete genomes, as represented by multiple copies of *dsrAB* and *aprAB* (Umezawa et al., 2016). The members of the family *Acidiferrobacteraceae* have been frequently detected in a variety of environments (Dyksma et al., 2016; Thouin et al., 2019; Wu et al., 2021), but only two assemblies of *Af. thiooxydans* were observed in the NCBI database. The restricted genome information limited the access to their genomic features in depth, which could give a deep understanding about their environmental adaptations.

Bioinformatic analyses highlighted the potential roles of various integrative mobile genetic elements (MGEs) in promoting the evolutionary adaptation of bacteria (Leal et al., 2020). Among the different types of MGEs, insertion sequences (ISs) contained a single open reading frame (ORF) encoding the transposase and inverted repeats at both ends. ISs transposition directly brought about gene deletion, inversion, and even rearrangement, which impacted a variety of bacterial life processes such as drug resistance, virulence, and metabolism (Vandecraen et al., 2017). The clustered regularly interspaced short palindromic repeats (CRISPR)/Cas (CRISPR-associated proteins) system could integrate fragments of foreign nucleic acids into CRISPR arrays and embed in microbial genomes, achieving immunity for virus defense purposes (Faure et al., 2019). Prophages, namely, integrated virus genomes, were also common MGEs, which might be conducive to recruiting novel functionalities (Ramisetty and Sudhakari, 2019). Genomic islands (GIs) were mostly derived from horizontal gene transfer (HGT), which were the large fragments that enriched functional genes (Dobrindt et al., 2004). It was apparent that various MGEs played crucial roles in bacterial genome evolution and adaptation to specific environmental stress.

We isolated the strain *Af. thiooxydans* ZJ from Zijinshan Copper Mine, Fujian Province, China. The complete genome record CP080624 has been uploaded to NCBI that refreshed its draft genome (MDCF00000000.1). This study is the first time to report the complete genome of *Af. thiooxydans*. Experimental results have revealed that ZJ could adapt to a wide range of pH and temperature. However, there were many unknowns about the important MGEs that assisted the unique *Acidiferrobacteraceae* to acclimatize to the extreme acid environment. Further evidence of the adaptative mechanisms was urgently needed, especially supported by a complete genome in depth. In this study, the unique molecular taxonomy of *Af. thiooxydans* ZJ was confirmed by using orthoANI algorithm and pan-genome analysis. The MGEs in the single chromosome, such as IS, CRISPR, prophage, and GI, were identified to access its acidic environmental adaptation.

## Materials and Methods

### Isolation and Cultivation

*Acidiferrobacter thiooxydans* ZJ was isolated from the AMD sample of Zijinshan Copper Mine (25°10′41″ N ∼ 25°11′44″ N, 116°24′00″ E ∼ 116°25′22″ E), China. The liquid samples from effusion pool were inoculated in 9K medium (pH 1.6) supplemented with 22.4 g/L ferrous sulfate and 5 g/L elemental sulfur. They were cultivated at 40°C in a rotary shaker at 170 rpm. The 9K medium contained the following components (g/L): (NH_4_)_2_SO_4_ (3), K_2_HPO_4_ (0.5), KCl (0.1), Ca(NO_3_)_2_ (0.01), and MgSO_4_⋅7H_2_O (0.5). Strains were purified by serial gradient dilution and identified by 16S rRNA amplification and sequencing. According to the result from NCBI nucleotide collection (nr/nt) blast, the type strains provided by LPSN (List of Prokaryotic names with Standing in Nomenclature) (Parte, 2018) were employed to construct the phylogenetic tree using the maximum likelihood method by Molecular Evolutionary Genetics Analysis 11 (MEGA, v11) (Tamura et al., 2021). Bootstrap analysis was carried out on 1,000 replicate input data sets.

### DNA Extraction and Sequencing

Cells of *Af. thiooxydans* ZJ were harvested by centrifugation (12,000 *g* for 10 min at 4°C) after cultivating to late exponential phase. Total DNA was extracted using the QIAamp DNA mini kit (Qiagen, Hilden, Germany) according to the manufacturer’s instructions. Purified genomic DNA was quantified by a TBS-380 fluorometer (Turner BioSystems Inc., Sunnyvale, CA, United States). The DNA with high quality (OD260/280 = 1.8–2.0, > 10 μg) was sequenced with a combination of PacBio RS and Illumina HiSeq platform supported by Shanghai Majorbio Bio-pharm Technology Co., Ltd. (Shanghai, China).

### Genome Assembly and Annotation

The reads generated from PacBio and Illumina platform were filtered to form clean data. Then, the clean data were assembled into a contig using the hierarchical genome assembly process (HGAP) and Canu with default parameters (Chin et al., 2013; Koren et al., 2017). The last circular step was checked and finished manually, generating a complete genome of seamless chromosome. Finally, error correction of the PacBio assembly results was performed using the Illumina reads using Pilon (Walker et al., 2014). All of the analyses were performed using the free online platform of Majorbio Cloud Platform^1^.

Identification of ORFs was conducted using Glimmer v3.02^2^ (Delcher et al., 2007). ORFs less than 300 bp were discarded. Then, the remaining ORFs were queried against the databases of NCBI-nr, Swiss-Prot^3^, KEGG^4^, and COG^5^ to do functional annotation by blastp 2.2.28 +. Moreover, the genome annotation was performed using the Rapid Annotation Subsystem Technology (RAST) server^6^ (Aziz et al., 2008). In addition, tRNAs were identified by the tRNAscan-SE (v1.23^7^) (Chan et al., 2021) and rRNAs were determined by the Barrnap v0.9^8^.

### Comparative Genomics Analysis

Based on the 16S rRNA phylogenetic tree, the complete genomes of *Sulfurifustis variabilis* skN76, *Sulfuricaulis limicola* HA5, *Ectothiorhodospira* sp. BSL-9, and *Thioalkalivibrio sulfidiphilus* HL-EbGr7 from *Acidiferrobacteraceae* and *Ectothiorhodospiraceae*, together with the complete genomes of *Acidithiobacillus ferrooxidans* ATCC 23270, *Acidithiobacillus ferrivorans* SS3, *Acidithiobacillus thiooxidans* ATCC 19377, and *Acidithiobacillus caldus* ATCC 51756 from *Acidithiobacillaceae*, were selected for comparative studies.

OrthoANI values among the strains from *Acidiferrobacter* and the two groups mentioned above were calculated based on whole-genome sequences. Heatmaps were plotted on the OAT: OrthoANI Tool (v0.93.1) subsequently (Lee et al., 2016). Average Amino acid Identity (AAI) was calculated on the online site^9^ (Rodriguez-R and Konstantinidis, 2014).

The genome sequences of the strains from the two groups were annotated with Prodigal (Hyatt et al., 2010). Based on the Bacterial Pan-genome Analysis tool (BPGA v1.3), the pan-genome and core-genome were estimated using the USEARCH program (v11.0.667) available in BPGA, with a 50% cutoff of sequence identity (Chaudhari et al., 2016).

### Mobile Genetic Elements Identification

Insertion sequence and transposases distributed over *Af. thiooxydans* ZJ genome were predicted and classified using the ISfinder platform^10^ based on online blast analysis with an E-value of 1e-10 (Siguier et al., 2006). The information about the position, family type, and copy number of all the ISs was obtained from the blast results. Functional regions around the ISs were identified and selected to generate a physical gene map.

The CRISPRCasFinder web tool^11^ (Couvin et al., 2018) was employed to identify the CRISPR/Cas array, including the repeat sequences and spacer sequences. The differences of the repeat sequences at each base site were visualized using WebLogo^12^ online server (Crooks et al., 2004). The RNA secondary structure of the repeat sequence was predicted by RNAfold^13^ (Mathews et al., 1999). The spacers were further annotated by blastn against the NCBI-nt database.

Integrated bacteriophages (prophages) prediction and the active evaluation were conducted using Prophage Hunter^14^ (Song et al., 2019). There are two alternative strategies: either incorporating similarity searches to increase accuracy or skipping it to raise the possibility of finding novel phages. In order to ensure the accuracy, we did not ignore the similarity matching in this study. Then, the qualified phages were classified taxonomically at the family and genus levels. The genome lengths of prophages from different families were calculated.

The GI was predicted by Island-viewer 4 online server^15^ (Bertelli et al., 2017) joining three methods: IslandPath-DIMOB (Hsiao et al., 2005), IslandPick (Langille et al., 2008), and SIGI-HMM (Waack et al., 2006). The proteins corresponding to GI predicted by at least one way from Island-viewer 4 online server were counted.

## Results and Discussion

### Genomic Features of *Acidiferrobacter thiooxydans* ZJ

**FIGURE 1:**
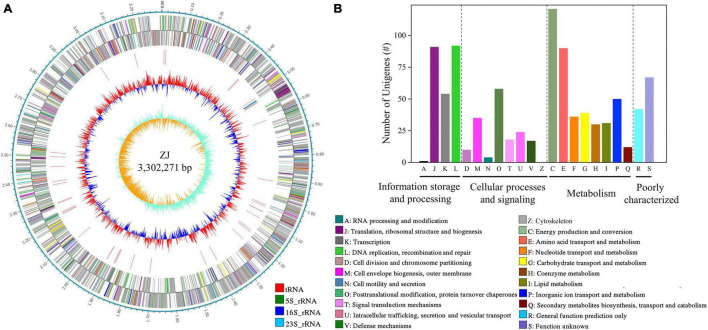
Circular map of *Af. thiooxydans* ZJ **(A)** and the classification of COG functions **(B)**. From outside to inside: rules for reads; CDS on sense DNA strand; CDS on antisense DNA strand; rRNA and tRNA; GC content; GC skew.

After filtering, data from PacBio and Illumina corresponded to an 81 × and 132 × coverage. The circular genome map of *Af. thiooxydans* ZJ is shown in Figure 1A. The genome has a single chromosome of 3,302,271 bp, with a GC content of 63.61%. There were 3,343 coding DNA sequences (CDSs), 46 tRNAs, and 3 rRNAs in the complete genome. The average gene length was 850 bp and the coding density (%) was 87.33%. In addition, 56 repeated sequences were observed with a total of 11,206 bp in length. There were 2,667 genes uncovered by NCBI-nr, KEGG, Swiss-Prot, GO, and COG databases, accounting for 78.65% of all the predicted genes. The COG annotation (Figure 1B) revealed that the genes belonging to C (energy production and conversion), E (amino acid transport and metabolism), J (translation, ribosomal structure, and biogenesis), and L (replication, recombination, and repair) ranked as the top four COG classifications.

**FIGURE 2:**
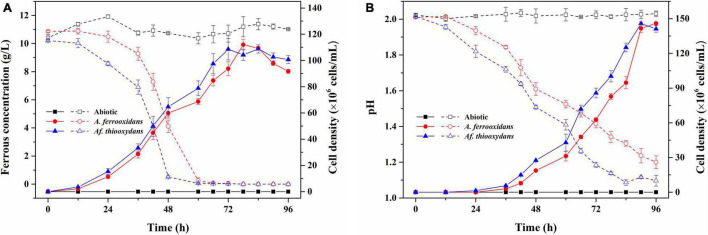
The growth and iron/sulfur oxidation rate of *Af. thiooxydans* ZJ and *A. ferrooxidans* ATCC 23270 under the same culture conditions (pH 2.0, Temperature 30°C). The dashed lines represent the ferrous concentration in the left *y*-axis and the solid lines represent the cell density in the right *y*-axis **(A)**. The dashed lines represent the pH in the left *y*-axis and the solid lines represent the cell density in the right *y*-axis **(B)**.

It has been reported that *Af. thiooxydans* could obtain energy by oxidizing ferrous iron, pyrite, element sulfur, sulfide, or tetrathionate in the oligotrophic habitat (Hallberg et al., 2011). As an efficient iron and sulfur oxidizer, *Af. thiooxydans* ZJ showed faster growth and oxidation rate of iron and sulfur than *A. ferrooxidans* ATCC 23270 under the same culture conditions (pH 2.0, Temperature 30°C, Figure 2), which was an essential individual of the microbial community in the bioleaching system (Wu et al., 2021). Herein, the genes encoding blue copper-containing protein rusticyanin and the sulfur-oxidizing gene cluster (*sox*) were identified in *Af. thiooxydans* ZJ genome, supporting the strong iron and sulfur oxidation capacity (Figure 2). Besides, the *recA*-dependent acid-tolerance system (COG0468), which was an inducible pathway involved in DNA repair, has been identified in *Af. thiooxydans* ZJ, indicating that *Af. thiooxydans* ZJ may present a flexible response and strong adaptation to the extremely acid environments based on recombination and repair, just like the common acidophiles in mining areas (Bellenberg et al., 2019; Ma et al., 2019).

### Phylogenetic Position of *Acidiferrobacter thiooxydans* ZJ Based on Comparative Genomics

**FIGURE 3:**
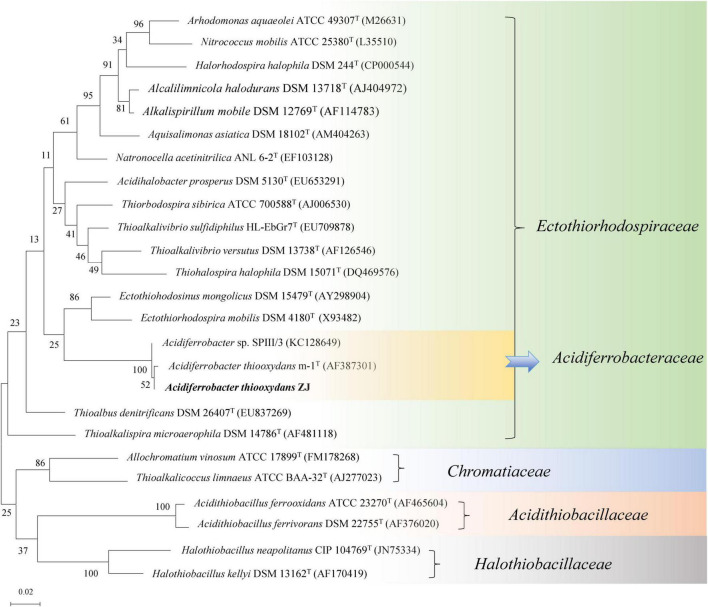
Phylogenetic tree of *Af. thiooxydans* ZJ with the neighboring standard species based on 16S rRNA sequences. The tree was constructed by maximum likelihood method with bootstrap values calculated from 1,000 trees. The numbers at each clustering node indicate the percentage of bootstrap supporting. The scale bar 0.02 indicates evolutionary distance.

According to the blast result of the NCBI-nr database, the nearby standard strains (25 strains) from LPSN were selected to construct a phylogenetic tree based on their 16S rRNA genes. As shown in Figure 3, the different families (-*aceae*) were well separated within the tree, and *Af. thiooxydans* ZJ formed a clade with *Af. thiooxydans* m-1 and *Acidiferrobacter* sp. SPIII/3. In addition, they were more closely related to *Ectothiorhodospira* and *Thioalkalivibrio* branches. However, they departed from the outgroup cluster formed by *A. ferrooxidans* and *A. ferrivorans*, which belonged to *Acidithiobacillaceae.* Only a few bacteria from the family *Ectothiorhodospiraceae* have been identified as iron oxidizers (Davis-Belmar et al., 2008; Hallberg et al., 2011). One was *Thiodictyon* sp. strain L7, which oxidized ferrous iron in anaerobic conditions; the other was *Acidihalobacter prosperus* (originally as *Thiobacillus prosperus*), which was capable of oxidizing both iron and sulfur compounds (Huber and Stetter, 1989; Pablo Cárdenas et al., 2015). This oxidation character also supported a separate status of *Acidithiobacillaceae* from *Ectothiorhodospiraceae*.

**FIGURE 4:**
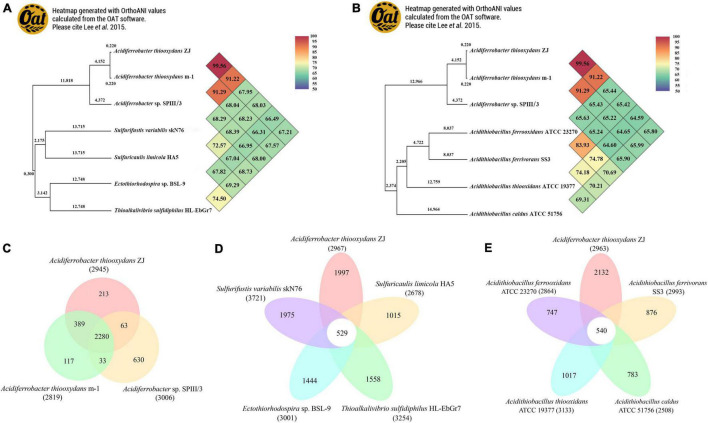
Heatmap generated with OrthoANI values calculated using the OAT software between *Acidiferrobacter* and other closely related species from *Ectothiorhodospiraceae* **(A)** and *Acidithiobacillaceae* **(B)**. The numbers at each clustering node indicate the branch length. Pan-Genome analysis among *Acidiferrobacter* **(C)**, *Af. thiooxydans* ZJ with *Ectothiorhodospiraceae* **(D)**, and *Acidithiobacillaceae* **(E)**.

Based on the 16S rRNA phylogenetic analysis results, we further calculated the OrthoANI distance among the nearby complete genomes of 11 strains. The genomic features of the selected genomes are summarized in Table 1. As shown in Figure 4, the strain *Af. thiooxydans* ZJ and *Af. thiooxydans* m-1 belonged to the same genomic species (OrthoANI value = 99.56%), but they were distinct from the genomic species of SPIII/3 (OrthoANI value = 91.22% and 91.29%). An OrthoANI value of 95–96% could be used as a cutoff for bacterial species demarcation, which corresponded to a cutoff point of 70% digital DNA–DNA hybridization (DDH) (Lee et al., 2016). The strains within the family maintained distant relationships with the similarity as low as 66.49–68.03% (Figure 4A). Recent studies have reassigned *Af. thiooxydans* to the new *Acidiferrobacteraceae* family with *Sulfurifustis variabilis* skN76 (Kojima et al., 2015) and *Sulfuricaulis limicola* HA5 (Kojima et al., 2016), but the OrthoANI between them were not higher than that with other members from *Ectothiorhodospiraceae*, such as *Ectothiorhodospira* sp. BSL-9 and *Thioalkalivibrio sulfidiphilus* HL-EbGr7. Meantime, as expected, the lower genomic relatedness with *Acidithiobacillus* was also confirmed by their greater distances with all the similarity less than 66% (Figure 4B). In addition, the calculation results of AAI (lower than 60%, Supplementary Table 1) also demonstrated that the genus *Acidiferrobacter* occupied a unique taxonomic position, which was distant from *Ectothiorhodospiraceae* and *Acidithiobacillaceae*.

**TABLE 1:** Overview of the complete genomes selected from *Ectothiorhodospiraceae* and *Acidithiobacillaceae*.

Species	Isolation source	Accession number	Assembly level	Genome size/Mb	GC%	Gene	Protein	rRNA	tRNA	References
*Acidiferrobacter thiooxydan*s ZJ	Zijinshan Copper Mine	CP080624.1	Complete	3.15	63.6	3,115	3,039	3	46	This study
*Acidiferrobacter thiooxydan*s m-1	Coal strip mine refuse	NZ_PSYR00000000.1	Contig	3.25	63.7	3,124	3,037	3	45	Issotta et al., 2018
*Acidiferrobacter* sp. SPIII/3	Mining area near to La Esperanza in Murcia, Spain	NZ_CP027663.1	Complete	3.40	64.2	3,414	3,295	3	45	Issotta et al., 2018
*Sulfurifustis variabili*s skN76	Sediment of a freshwater lake	NZ_AP014936.1	Complete	3.96	67.5	3,909	3,844	3	46	Umezawa et al., 2016
*Sulfuricaulis limicola* HA5	Sediment from a meromictic lake from residential area	NZ_AP014879.1	Complete	2.86	61.4	2,803	2,742	3	46	Umezawa et al., 2016
*Ectothiorhodospira* sp. BSL-9	Big Soda Lake, Nevada	NZ_AP014879.1	Complete	3.55	63	3,300	3,158	6	46	Hernandez-Maldonado et al., 2016
*Thioalkalivibrio sulfidiphlius* HL-EbGr7	Thiopaq bioreactor used to remove H_2_S from biogas	NC_011901.1	Complete	3.46	65.1	3,372	3,266	3	44	Muyzer et al., 2011
*Acidithiobacillus ferrooxidans* ATCC 23270	Acid, bituminous coal mine effluent	NC_011761.1	Complete	2.98	58.8	3,075	2,893	6	82	Valdés et al., 2008
*Acidithiobacillus ferrivorans* SS3	Sediment, Norilsk, Russia	NC_015942.1	Complete	3.21	56.6	3,200	3,038	6	47	Liljeqvist et al., 2011
*Acidithiobacillus thiooxidans* ATCC 19377	Kimmeridge clay	NZ_CP045571.1	Complete	3.42	53	3,573	3,388	6	76	Valdes et al., 2011
*Acidithiobacillus caldus* ATCC 51756	Coal spoil at Kingsbury Mine	NZ_CP026328.2	Complete	2.73	61.7	2,706	2,550	6	47	Tapia et al., 2012

Pan-genome analysis was performed to identify the corresponding core and dispensable genome. A core genome containing 2,280 CDSs was observed within *Acidiferrobacter*; meantime, *Af. thiooxydans* ZJ and m-1 shared 389 genes, while SPIII/3 harbored more unique genes (Figure 4C). Compared with the genomes from the broad *Ectothiorhodospiraceae*, *Af. thiooxydans* ZJ harbored 1,997 unique genes apart from other genomes (Figure 4D), implying a relatively high degree of genomic diversity and low relatedness with the other members of the family mentioned above. Meanwhile, there were 540 genes shared with *Acidithiobacillus*, and the strain-specific genes in *Af. thiooxydans* ZJ were as high as 2,132 among the selected *Acidithiobacillus* species (Figure 4E). Previous studies have demonstrated that the genetic traits concerning adaptation, resistance and virulence were more often governed by dispensable genome (Maistrenko et al., 2020); therefore, *Af. thiooxydans* ZJ was hypothetically conferred selective advantages and special adaptability to the similar extreme environment.

### Insertion Sequences and Transposases in *Acidiferrobacter thiooxydans* ZJ

**FIGURE 5:**
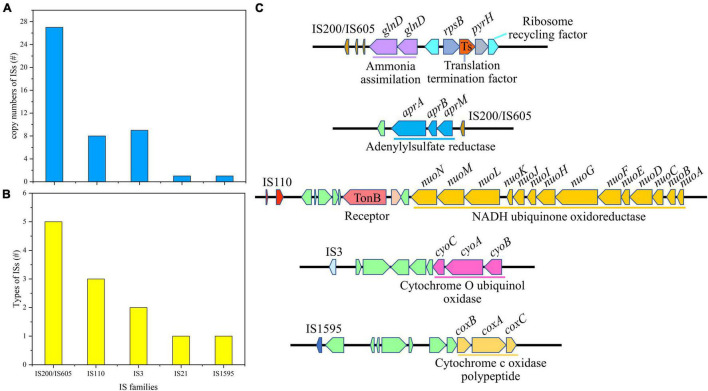
The copy number of insertion sequences **(A)**, the families of ISs **(B)**, and the putative laterally transferred regions contain ISs **(C)**.

Insertion sequences are the simplest type of MGEs in microbial genomes, which could be classified based on general characteristics of their DNA sequences and relevant transposases (Vandecraen et al., 2017). There were 12 types of ISs in *Af. thiooxydans* ZJ genome, belonging to 5 families with a total of 46 copies. The IS200/IS605 family, including ISAfe8, ISAba30, ISMex34, ISSoc9, and ISCARN6, exhibited the highest copy number of 27 in the genome (Figures 5A,B). It has been reported that IS200/IS605 was an ancient and stable IS family with the smallest known DNA transposases, working based on the so-called peel-and-paste mechanism (Barabas et al., 2008). The transposases in *Af. thiooxydans* ZJ genome could be categorized into three types, belonging to DEDD, DDE, and Y1 HUH (Supplementary Table 2). Unlike DDE ISs, ISs with DEDD or Y1 HUH transposases neither carry the terminal inverted repeats (IRs) nor generate flanking direct repeats (DRs) on insertion; meanwhile, Y1 HUH encoding ISs were able to form hairpin secondary structures particularly (Siguier et al., 2014). This secondary hairpin structure was formed to transposase specific recognition and catalyzed single-strand cleavage that could help stabilize the nuclear protein complex. We herein supposed that ISs of *Af. thiooxydans* ZJ may utilize a variety of strategies to shape the *Af. thiooxydans* ZJ genome effectively.

It was well acknowledged that ISs could mobilize neighborhood genes in certain cases. The adjacent gene compositions of ISs were examined in *Af. thiooxydans* ZJ. As shown in Figure 5C, IS200/IS605 was mainly located upstream of the gene (*glnD*) coding for ammonia assimilation along with the functional regions of translation termination and ribosome recycling. IS200/IS605 was also located downstream of the genes (*aprA*, *aprB*, and *aprM*) coding adenylylsulfate reductase. Adenylylsulfate reductase mainly utilized adenylate as a substrate and thioredoxin as an electron donor to reduce active sulfate to sulfite (Zhang et al., 2016). IS110 was flanked by the genes coding for NADH ubiquinone oxidoreductase, which was involved in electron transfer in the respiratory system. Besides, IS3 presented upstream of the genes (*cyoA*, *cyoB*, and *cyoC*) coding for cytochrome O ubiquinone oxidase, and IS1595 was located upstream of the genes (*coxB*, *coxA*, and *coxC*) coding for cytochrome C oxidase polypeptide (Figure 5C). All these enzymes are important oxidases involved in bacterial electron transfer (Dinamarca et al., 2002), providing essential functions for *Af. thiooxydans* ZJ to survive in the oligotrophic and stressful environment caused by heavy metals. Hence, the functional genes around these ISs may contribute to the processing of sulfide, assimilation of ammonia from the surroundings, and electron transfer, improving the favorable survival and strong adaptation of *Af. thiooxydans* ZJ in AMD environment.

### CRISPR/Cas Systems in *Acidiferrobacter thiooxydans* ZJ

The CRISPR/Cas systems were adaptive immunity systems that served as viral and plasmid defense mechanisms developed by bacteria and archaea (Faure et al., 2019). The *Af. thiooxydans* ZJ genome harbored a quite large CRISPR array (13,779 bp), which contained 230 direct repeats (28 bp each) separated by 229 different spacers of similar sizes (32–36 bp) (Supplementary Table 3), suggesting that it was fairly defensive to the foreign genetic elements along the evolutionary process (Kojima et al., 2015). Based on the *cas* gene arrangement and the presence of Cas3 (Cas1-Cas6-Csy3-Cas3-Cas2), the CRISPR/Cas system of *Af. thiooxydans* ZJ could be classified as subtype I-F; additionally, type I CRISPR/Cas systems were speculated to directly target DNA against invading viruses (Makarova et al., 2015). Interestingly, our results of blastn program against the NCBI-nt database showed that the spacer 111 (ATTATGACGCGCTGGCACGAGGATGATCTCGT) has a similarity of 100% to a fraction genome of the *Myoviridae* sp. isolate ct6gw13. *Myoviridae* has been reported to play a major part in viral populations in the sulfidic mine tailings (Gao et al., 2020), while further studies regarding viral diversity and functions in the acid mine environment were needed to expand the knowledge.

The formation of secondary structure of CRISPR repeats was closely related to its stability and mechanism. If the predicted secondary structure was mainly formed by “loop,” the interaction between spacer and foreign DNA was performed by a single “repeat-spacer” unit during the transcription process (Kunin et al., 2007). The repeats may serve to mediate the coupling cleavage of exogenous genetic materials and Cas-encoded proteins. Otherwise, the contact between spacers and foreign DNA was assisted by two adjacent repeats (Kunin et al., 2007). As shown in Supplementary Figure 1, the 28-bp repeats in *Af. thiooxydans* ZJ were rather conservative. Only at positions 15, 16, and 19 did the site conservation fluctuate slightly. What is more, the stem length of the proposed RNA secondary structure of repeat sequence was 8 bp, that is to say, the stem-loop structure was dominated by “stem.” Thus, it could be speculated that the defense function of the spacer against the foreign DNA invasion may be facilitated through the complementation of two adjacent repeats (Kunin et al., 2007).

In short, *Af. thiooxydans* ZJ has evolved a unique I-F CRISPR/Cas system, which may have resisted the intrusion of foreign viruses, attaching great significance for its adaptation and development in the extremely acidic environment.

### Prophages in *Acidiferrobacter thiooxydans* ZJ

Bacteriophage, as a source of foreign DNA, are increasingly recognized to contribute to gene flow in prokaryotes, which also led to bacterial genome plasticity (Dobrindt et al., 2010). Prophages played prominent roles in the host-adaptive traits and genetic diversification by delivering functional genes among different strains (Ramisetty and Sudhakari, 2019). Based on the score of each candidate, regions with scores higher than 0.8 were defined as an “active” prophage; the score of an “ambiguous” one ranged from 0.5 to 0.8, and a score lower than 0.5 was defined as an “inactive” one (Song et al., 2019). In the genome of *Af. thiooxydans* ZJ, 85 prophage candidates were identified, of which three candidates were the ambiguous ones. None of these phages was predicted to be active (Supplementary Table 4). A total of 1,807 fragments were obtained, including CDS information and functional annotation results based on NCBI-nr, Pfam, and InterPro databases (Supplementary Table 5). These prophage genes were mainly related to encoding phage structure and assembly proteins (e.g., head–tail protein, tape measure protein), or the enzymes that play vital roles in the whole process of phage infection (e.g., transposase, site-specific DNA recombinase, and integrase). There was a major category of membrane-associated protein structures named ATP binding cassette (ABC) transporters, which are involved in the nutrient uptake through medium- and high-affinity pathways in bacteria (Locher, 2016). In this study, it was estimated to be mainly involved in the transport of phosphate or peptides. Meanwhile, genes encoding TonB-dependent receptors have been identified, which participated in the uptake of rare earth metals (Noinaj et al., 2010; Ochsner et al., 2019), membrane homeostasis (Menikpurage et al., 2019), and secretion of proteins (Gómez-Santos et al., 2019). Moreover, many genes encoding hydrolases, diguanylate cyclase, peptidases, and the toxin–antitoxin (TA) system have also been observed, which may improve the survival and growth of *Af. thiooxydans* ZJ (Costa et al., 2018).

**FIGURE 6:**
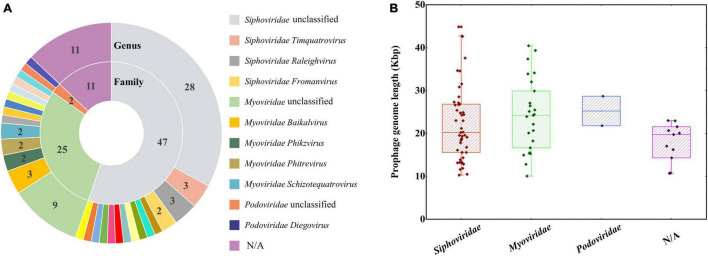
The closest phages classified according to family and genus levels **(A)**. The numbers represented the closest phages belonging to the different families. A boxplot of prophage genome lengths based on closest phages family taxonomy **(B)**.

The closest phage relatives were also identified, of which 74 prophage genome regions mainly belonged to the family *Siphoviridae* (47, with a percentage of 55.29%), *Myoviridae* (25, with a percentage of 29.41%), and *Podoviridae* (2, with a percentage of 2.35%) (Figure 6A and Supplementary Table 6). All the three families were affiliated to the order *Caudovirales*, which were the tailed double-stranded DNA bacteriophages, including 14 families and 927 genera (Lefkowitz et al., 2018). The length of prophage fragments was presented in Figure 6B. The result was consistent with the most common phages in nature (Ackermann, 2007).

### Genomic Islands in *Acidiferrobacter thiooxydans* ZJ

**FIGURE 7:**
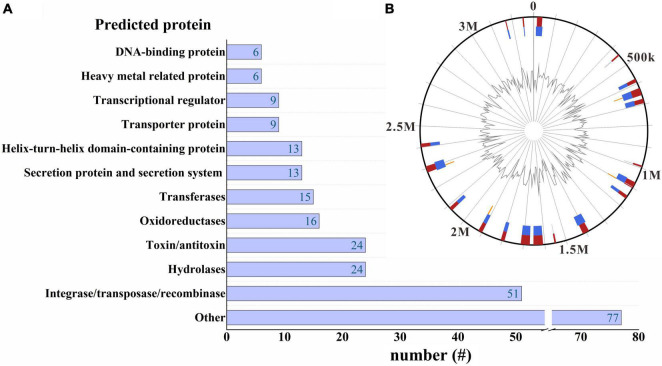
A horizontal bar graph of the predicted proteins located on the GI regions **(A)**. GI distribution in *Af. thiooxydans* ZJ genome **(B)**. The blue and orange lines represent the GI predictions by IslandPath-DIMOB and SIGI-HMM, respectively, and the red line in the outer circle represents the comprehensive results by different methods. The peak map in the inner circle represents the variation of the GC content.

GIs are commonly believed to be relics of HGT and clusters of genes encoding different functions, which plays a role in genome plasticity and evolution, offering a selective advantage for host bacteria (Dobrindt et al., 2004; Juhas et al., 2009). A total of 23 GIs were identified in the *Af. thiooxydans* ZJ genome ranging from 4.164 kb to 44.743 kb in size (Figure 7B). There were 415 predicted genes located in these GIs, among which 152 genes were annotated as “hypothetical proteins” with unknown functions. The annotated proteins were mostly related to integrase/transposase/recombinase (51 genes) and antitoxin/toxin (24 genes) (Figure 7A). In addition, some GIs also contained the genes encoding proteins for maintaining bacterial survival, metabolism, and growth (e.g., hydrolases, secretion system, and oxidoreductase). These strongly evidenced that GIs in *Af. thiooxydans* ZJ genome helped transfer a large number of gene families to the host bacteria to improve its adaptability in high metal concentration environment.

Intriguingly, integrases, transposases, and recombinases were powerful tools for insertion mutations, which also played a critical role in shaping the genome and events leading to speciation (Kazazian, 2004). There are 29 genes in the GI region involved in encoding IS family transposases (IS3, IS21, ISL3, etc.), promoting the movement of DNA segments to new locations within and between genomes. The majority of the potential GIs were noted to flank by these genes, suggesting that transposases may be associated with the acquisition of GIs (Rao et al., 2020; Supplementary Table 7).

It was noteworthy that the TA system was widely distributed in prokaryotes, which play a wide range of biological functions, including plasmid addiction, antibiotic tolerance, and defense against phages (Otsuka, 2016; Harms et al., 2018). Many genes regarding bacterial toxicity have been found in the GIs of *Af. thiooxydans* ZJ, such as the genes encoding RelE/ParE family toxin, VapC family toxin, Phd/YefM family antitoxin, and addiction module protein. Most of them belonged to the type II TA system, which neutralized toxicity by forming a protein–protein complex between antitoxin and toxin (Unterholzner et al., 2013). Previous studies have demonstrated that the TA system was involved in the stabilization of large genomic segments (Szekeres et al., 2007) and its addictive properties allowed them to integrate stably in bacterial chromosomes (Leplae et al., 2011). We hypothesized that GIs might play an essential role in the evolution of virulence in *Af. thiooxydans* ZJ. Meanwhile, genes encoding heavy metal-responsive transcriptional regulator and heavy metal-binding domain-containing protein have also been identified in GIs. Combined with previous surveys of *Acidithiobacillus* (Hemme et al., 2016; Li et al., 2019), the influence of HGT on the evolution of heavy metal resistance in *Af. thiooxydans* ZJ surviving in extreme environments needed to be further investigated.

## Conclusion

**FIGURE 8:**
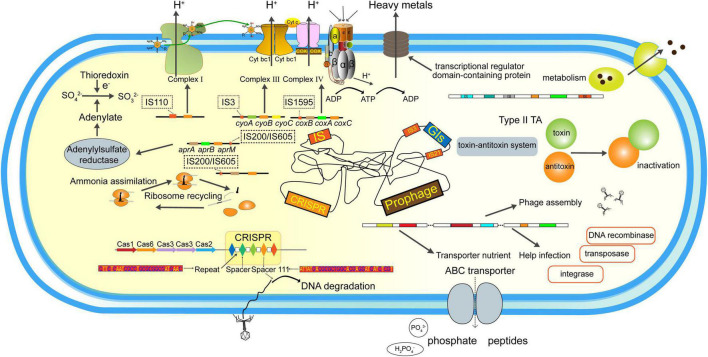
Functional repertoires of the *Af. thiooxydans* ZJ. The MGEs and their predicted gene products identified in this study.

In the present study, the iron and sulfur oxidizer *Af. thiooxydans* ZJ was isolated from AMD, yielding a complete genome with 3,302,271 bp with a high GC content of 63.61%. Comparative genomic analysis revealed that *Af. thiooxydans* ZJ was distinctive within the *Acidiferrobacteraceae* family phylogenetically, while it was more closely related to *Acidithiobacillaceae* physiologically. Subsequently, a considerable various repertoire of MGEs were assessed, including 5 families of ISs, an I-F subtype CRISPR/Cas system, 85 prophage regions, and 23 GIs generally (Figure 8). ISs may harbor different insertion patterns based on transposases. Most GIs were also annotated to contain the genes encoding integrase/transposase/recombinase that facilitate the insertion and integration of external fragments. The blast results of CRISPR spacers suggested that it may resist phage intrusion, which was also supported by the discovery of prophages. The prophage regions encoded functional enzymes beneficial for the growth probably fought and derived by *Af. thiooxydans* ZJ. Collectively, genome plasticity regarding the gain/loss of MGEs contributed to the environmental adaptation of *Af. thiooxydans* ZJ in extremely acid niche with high metal concentrations. The effects of MGEs on bacterial genome shaping and adaptation mechanisms deserved further explorations extensively and deeply. Meanwhile, more experiments about the relevant genes are needed to verify the effectiveness.

## Data Availability Statement

The datasets presented in this study can be found in online repositories. The names of the repository/repositories and accession number(s) can be found in the article/Supplementary Material.

## Author Contributions

LM, WY, and XL: conceptualization. WY, SH, RL, and HL: methodology. XH and JX: validation. WY, XH, and JX: formal analysis. LM and WY: investigation and writing—original draft preparation. LM and XL: resources, supervision, and project administration. LM: data curation. SH, RL, HL, and XL: writing—review and editing. All authors have read and agreed to the published version of the manuscript.

## Conflict of Interest

The authors declare that the research was conducted in the absence of any commercial or financial relationships that could be construed as a potential conflict of interest.

## Publisher’s Note

All claims expressed in this article are solely those of the authors and do not necessarily represent those of their affiliated organizations, or those of the publisher, the editors and the reviewers. Any product that may be evaluated in this article, or claim that may be made by its manufacturer, is not guaranteed or endorsed by the publisher.

## Footnotes

1
www.majorbio.com

2
http://ccb.jhu.edu/software/glimmer/index.shtml

3
http://uniprot.org

4
http://www.genome.jp/kegg/

5
http://www.ncbi.nlm.nih.gov/COG

6
http://rast.nmpdr.org

7
http://lowelab.ucsc.edu/tRNAscan-SE

8
github.com/tseemann/barrnap

9
http://enve-omics.ce.gatech.edu/aai/

10
https://www-is.biotoul.fr/

11
https://crisprcas.i2bc.paris-saclay.fr

12
http://weblogo.threeplusone.com/

13
http://rna.tbi.univie.ac.at/cgi-bin/RNAWebSuite/RNAfold.cgi

14
https://pro-hunter.bgi.com/

15
https://www.pathogenomics.sfu.ca/islandviewer/
